# Supplementary material for: Conformational Disorder Analysis of the Conditionally Disordered Protein CP12 from Arabidopsis thaliana in Its Different Redox States
Source: Int J Mol Sci. 2023 May 26;24(11):9308. doi: 10.3390/ijms24119308 (PMC10253348; doi:10.3390/ijms24119308)
Supplement: Supplementary file 1 [file ijms-24-09308-s001.zip › ijms-2365413-supplementary.pdf]

Supporting Information

Conformational disorder analysis of the conditionally disordered protein CP12 from *Arabidopsis thaliana* in its different redox states

Alessandra Del Giudice[1], Libero Gurrieri[2], Luciano Galantini[1], Silvia Fanti[3], Paolo Trost[2], Francesca Sparla[2]*, Simona Fermani[3,4]

[1]Department of Chemistry, Sapienza University of Rome, Rome, Italy

[2]Department of Pharmacy and Biotechnologies, University of Bologna, Bologna, Italy

[3]Department of Chemistry "G. Ciamician", University of Bologna, Bologna, Italy

[3]Interdepartmental Centre for Industrial Research Health Sciences & Technologies, University of Bologna, Bologna, Italy

* Correspondence:
Corresponding Author
francesca.sparla@unibo.it

Keywords: Small angle X-ray scattering (SAXS); intrinsically disordered protein; conditionally disordered protein; post-translational modification; redox regulation; structural characterization; ensemble optimization; unstructured biology.

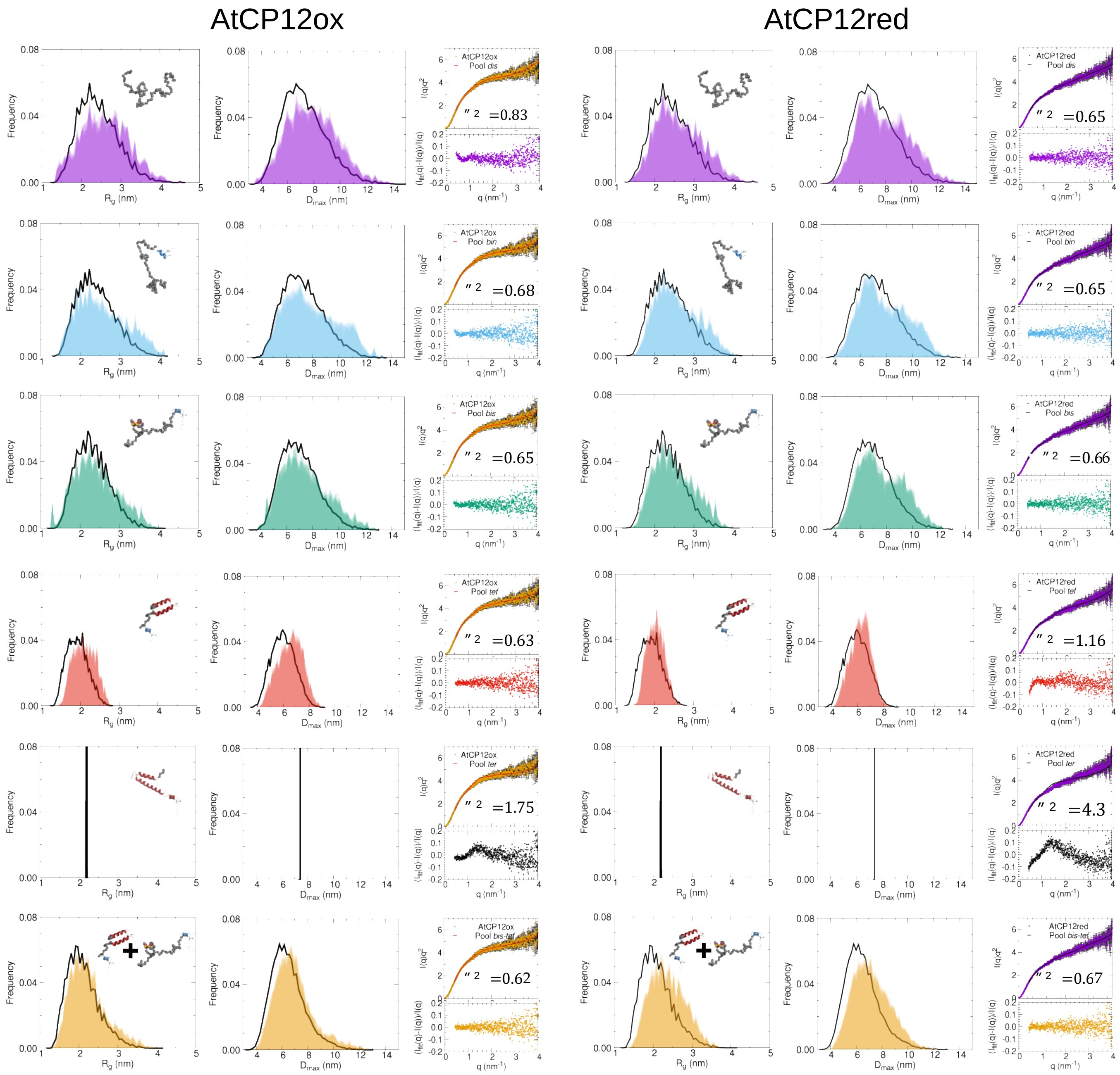

Figure S1. EOM optimizations. Results of the EOM optimizations to fit the SAXS profile of oxidized (left; AtCP12ox) and reduced (right; AtCP12red) AtCP12. For each pool of conformers (*dis* in purple, *bin* in light blue, *bis* in green, *tef* in red, *ter* in black, *bis-tef* in orange), the plots in the first and second columns of panels show R_g and D_{max} frequency distributions for the whole generated initial pool (black solid lines) and for the final selected ensembles to fit SAXS data (coloured shaded areas, overlap of the final distributions from eight independent optimizations). The pool codes reported are explained in the main text and in Figure 2, and here reminded by visualization of an example model. In the last column of panels the theoretical profile (solid line) obtained by the optimization with the lowest χ^2 among the repetitions is shown superimposed to the experimental data points (coloured dots) in Kratky plot representation, and below the corresponding normalized residuals $\frac{I_{calc}(q)-I_{exp}(q)}{I_{exp}(q)}$ are shown.

```
CrCP12              4 SGQPAVDLNKKVQDAVKEAEDACAKG-TSADCAVAWDTVEELSAAVSHKKDAV      52
                         |...::..|:.::|||::.||.. .|.:|..|||.|||||||.||.:|..
AtCP12              2 --APEGGISDVVEKSIKEAQETCAGDPVSGECVAAWDEVEELSAAASHARDKK      51

CrCP12             53 KADVTLTDPLEAFCKDAPDADECRVYED      80
                      |||.:  ||||.:|||.|:.:|||.|::
AtCP12             52 KADGS--DPLEEYCKDNPETNECRTYDN      77
```

Figure S2. Sequence alignment between AtCP12 and CrCP12. The alignment was performed with EMBOSS Matcher highlighting the N-terminal (in red) and C-terminal (in blue) possibly structured regions assumed when building conformational pools.

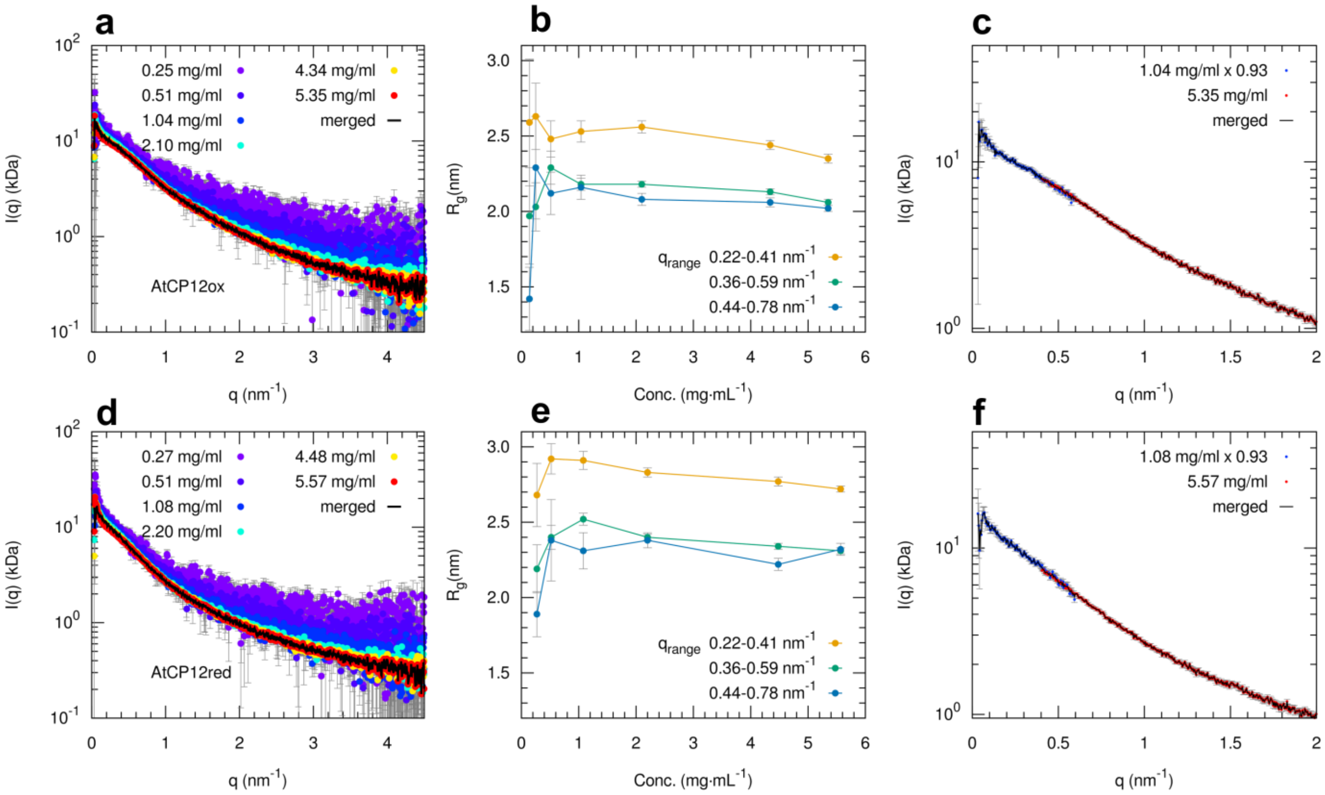

Figure S3. Experimental SAXS data collected for the dilution series of samples of oxidized and reduced AtCP12. Dilution series of oxidized AtCP12 (**a**) and reduced AtCP12 (**d**). The values of R_g estimated by Guinier fit in different q ranges for all concentrations are reported in (**b**) for oxidized AtCP12 and (e) for reduced AtCP12. The final merged data (black solid lines in (**a**) and (**d**)) are also shown in (**c**) and (**f**) together with the portion of the original experimental data at low (blue line) and high (red line) concentration.

Table S1. Summary of model independent SAXS data analysis of oxidized and reduced AtCP12.

Guinier fit	*Oxidized AtCP12*	*Reduced AtCP12*
R_g (Å)	2.02	2.32
$\sigma(R_g)$ (Å)	0.03	0.07
I(0) [kDa]	9.77	9.98
σ(I(0))	0.11	0.26
q range (nm^{-1})	0.44-0.65	0.44-0.57
Fidelity	1.0	0.92
Indirect Fourier transform		
R_g (nm)	2.29	2.9
$\sigma(R_g)$ (nm)	0.03	0.1
I(0) [kDa]	10.4	11.5
σ(I(0))	0.1	0.3
q range (nm^{-1})	0.36-3.5	0.36-3.5
D_{max} imposed for P(r) (nm)	9	11.6
D_{max} variability estimate (nm)	2	3
GNOM quality estimate	0.565	0.547
MW Bayesian		
q minimum (nm^{-1})	0.27	0.44
estimate (kDa)	7.3	7.3
estimate probability (%)	99.05	90.23
credibility interval (kDa)	6.65-9.05	6.65-9.05
interval probability (%)	99.35	99.62
MW from sequence (kDa)	8.26	8.26

Table S2. Summary of the EOM analysis of AtCP12 SAXS experimental data. (a) average structural parameters of the generated random pools of conformers: the radius of gyration (R_g), the maximum particle size (D_{max}), and the flexibility index (R_{flex}). The R_{flex} index (between 0 and 1) is a metric obtained from the entropy (information content) of the distribution and can be used to approximate the flexibility [37]. Using this metric the size distributions of the selected ensembles after fitting can be numerically compared to that of the initial random pool, which is supposed to represent the reference for the randomness of the distribution under the specific structural constraints. The average R_g of each pool is also compared to the experimental R_g obtained from the Guinier fit and reported as a % relative deviation. (b) Structural parameters and fitting results obtained by eight repetitions of the fitting algorithm applied to the SAXS data of AtCP12 in oxidized form (top) and in reduced form (bottom) considering each of the generated pools. The average value and standard deviation for each quantity are reported reported. From left to right, for each pool these parameters are shown: average R_g of the selected ensemble of conformers; average D_{max} of the selected ensemble of conformers; flexibility parameter of the selected distribution; check parameter representing the ratio between the standard deviations of the selected and starting size histograms distributions; experimental vs. calculated SAXS intensity agreement index χ^2; optimized constant background subtracted from the data while fitting.

(a)

**Pool**	**Pool average**		**Flexibility index**	**%R_g deviation**	
	**R_g (nm)**	**D_{max} (nm)**	***R_{flex}***	**Oxidized (R_g = 2.03 nm)**	**Reduced (R_g = 2.32 nm)**
dis	2.46	7.41	0.86	-21	-6
bin	2.43	7.39	0.88	-20	-5
bis	2.35	7.11	0.85	-16	-1
tef	1.94	6.06	0.89	5	17
ter	2.20	7.41	0.30	-8	5
bis-tef	2.14	6.59	0.84	-5	8

(b)

**Fitting data**	**Selected ensemble average**				**Flexibility index**		**Distribution width ratio**		**Goodness of fit**		**Constant background**	
	**$\langle R_g \rangle$ (nm)**	**σ**	**$\langle D_{max} \rangle$ (nm)**	**σ**	**$\langle R_{flex} \rangle$**	**σ**	**$\langle R_\sigma \rangle$**	**σ**	**$\langle \chi^2 \rangle$**	**σ**	**$\langle bg \rangle$ (kDa)**	**σ**
Oxidized AtCP12												
dis	2.61	0.04	8.05	0.17	0.91	0.001	1.21	0.005	0.85	0.017	0.12	0.007
bin	2.57	0.05	8.00	0.13	0.93	0.001	1.32	0.005	0.69	0.007	0.14	0.007
bis	2.44	0.04	7.54	0.14	0.91	0.001	1.30	0.007	0.66	0.007	0.14	0.006
tef	2.13	0.01	6.62	0.04	0.90	0.001	1.04	0.005	0.63	0.002	0.16	0.003
ter	2.20	0.00	7.41	0.00	0.23	0.002	0.73	0.008	1.65	0.004	0.14	0.001
bis-tef	2.28	0.03	7.05	0.12	0.86	0.001	1.15	0.008	0.63	0.001	0.15	0.004
Reduced AtCP12												
dis	2.69	0.03	8.14	0.17	0.90	0.001	1.18	0.005	0.66	0.001	0.121	0.004
bin	2.64	0.02	8.07	0.11	0.91	0.001	1.15	0.005	0.66	0.001	0.135	0.003
bis	2.56	0.02	7.75	0.11	0.89	0.001	1.18	0.007	0.66	0.001	0.128	0.004
tef	2.06	0.01	6.30	0.07	0.84	0.001	0.81	0.005	1.18	0.013	0.193	0.003
ter	2.20	0.00	7.41	0.00	0.23	0.003	0.73	0.007	4.35	0.013	0.171	0.001
bis-tef	2.44	0.02	7.38	0.10	0.86	0.001	1.07	0.005	0.68	0.002	0.142	0.002

Table S3. Summary of SAXS data acquisition information, sample details, and data analysis software used.

(a) Sample details for the SAXS experiments		
	Oxidized AtCP12	Reduced AtCP12
Concentration range (mg ml^{-1})	0.25-5.35	0.27-5.57
Storage and dilution buffer composition	25 mM K-phosphate, pH 7.5	25 mM K-phosphate, pH 7.5
Extinction coefficient for concentration determination from UV absorbance at 280 nm ($M^{-1}cm^{-1}$)	8730	8480

(b) SAXS data collection parameters	
Source, instrument	ESRF, BM29
Detector	PILATUS 1M
Beam geometry (mm^2)	0.7 x 0.7
Wavelength (Å)	0.9919
sample-to-detector distance (m)	2.872
q-measurement range (nm^{-1})	0.05-0.45
Absolute scaling method	water scattering I(0)= 0.01632 cm^{-1},<br>protein partial specific volume 0.735 cm^3 g^{-1}
Capillary path length (mm)	1.8
Injection volume (µl)	60
Exposure time (s)	1
Number of exposures	10
Extra flow time (s)	10
Sample temperature (ºC)	4

(c) Software employed for SAS data reduction, analysis, and interpretation	
Solvent subtraction, averaging and basic analysis (Guinier fit, P(R), MW)	ATSAS 3.2
Theoretical intensity calculations	CRYSOL 2.8
Ensemble optimization	EOM 3.0
Molecular graphics	PyMOL 1.8
